# Successful treatment of low‐risk myelodysplastic syndrome‐related anemia in patients with chronic kidney disease with daprodustat: A report of two cases

**DOI:** 10.1002/jha2.1057

**Published:** 2024-11-25

**Authors:** Hiroyoshi Kunimoto, Takayuki Sakuma, Takuma Ohashi, Mayoko Shirafuta, Hiroshi Teranaka, Hideaki Nakajima

**Affiliations:** ^1^ Department of Stem Cell and Immune Regulation Yokohama City University Graduate School of Medicine Yokohama Japan

**Keywords:** anemia, case report, daprodustat, MDS

## Abstract

Anemia is a major clinical manifestation seen in myelodysplastic syndromes (MDS). Treatment options for anemia in low‐risk MDS are limited. Especially, oral medication which is uniformly effective for anemia in low‐risk MDS is required. Hypoxia‐inducible factor (HIF) prolyl hydroxylase (HIF‐PH) inhibitors, such as daprodustat, are oral tablets effective for renal anemia. Pharmacological restoration of HIF activity by HIF‐PH inhibitors may be beneficial for MDS‐related anemia as well. Yet, their efficacy and safety against low‐risk MDS are unclear. Here, we report two cases of low‐risk MDS complicated with chronic kidney disease whose anemia responded to daprodustat treatment.

## 1 INTRODUCTION

Myelodysplastic syndromes (MDS) are myeloid malignancies in which cytopenias are the major clinical manifestation. More than 90% of MDS patients suffer from anemia [1, 2]. Treatment options for anemia in low‐risk MDS are limited. Erythropoietin (EPO) stimulating agents (ESA) often lose their effectiveness within 12 months [3]. MDS patients with short disease duration or low transfusion burden may respond to immunosuppressive therapies such as cyclosporin A (CsA) or prednisolone, albeit in only a subset of cases [4]. Although lenalidomide can reduce red blood cell (RBC) transfusion burden in 67% of MDS with del(5q), reduction of RBC transfusion can be achieved in only 25% of non‐del(5q) MDS cases by lenalidomide [5, 6]. Luspatercept can also improve anemia and reduce RBC transfusion burden in ESA‐naïve low‐risk MDS [7]. However, luspatercept is an injection agent which is administered subcutaneously. As such, oral medication which is uniformly effective for anemia in low‐risk MDS is required.

Hypoxia‐inducible factor (HIF) prolyl hydroxylase (HIF‐PH) inhibitors, such as daprodustat, are oral tablets used for renal anemia [8, 9]. Mechanistically, HIF‐PH inhibitors restore HIF activity thereby inducing transcription of genes encoding EPO and EPO receptor, improving anemia [10]. Therefore, these oral agents may be beneficial for anemia in low‐risk MDS. Yet, their exact efficacy and safety against low‐risk MDS are elusive. Here, we report two cases of low‐risk MDS complicated with chronic kidney disease (CKD) whose anemia responded to daprodustat treatment.

## 2 CASE PRESENTATION

**FIGURE 1:**
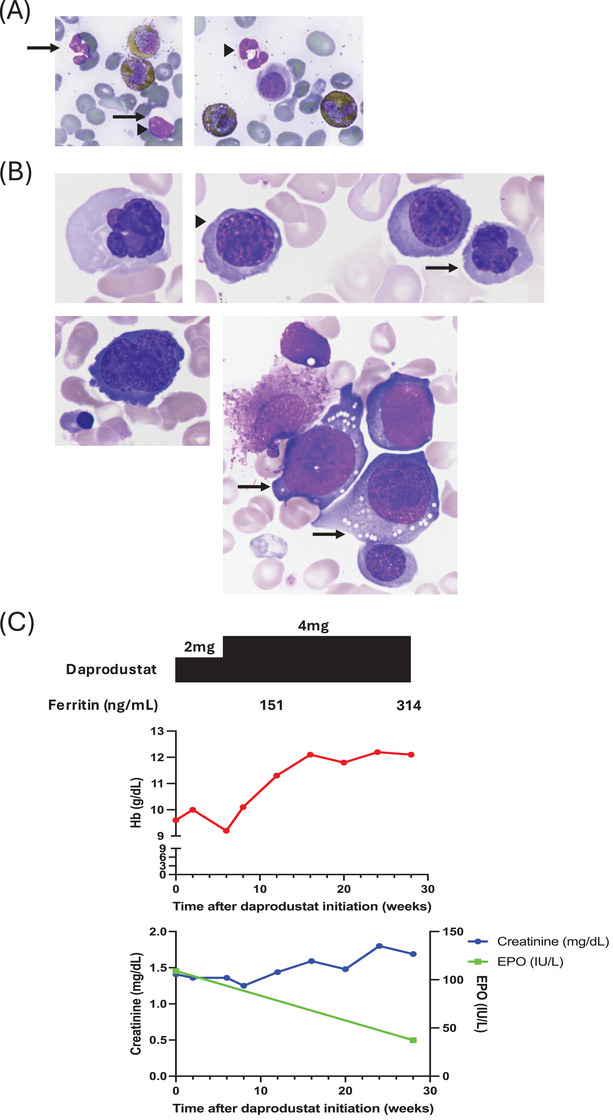
(A) Representative images of BM smears showing granulocytic dysplasia (left panel; decreased granules and nuclear hyposegmentation, right panel; nuclear hyposegmentation) of case #1. Solid arrows depict decreased granules. Arrowheads depict nuclear hyposegmentation. (B) Representative images of BM smears showing erythroid dysplasia (left upper panel; nuclear budding, right upper panel; nuclear budding (arrow) and karyorrhexis (arrowhead), left lower panel; multinuclearity, right lower panel; megaloblastoid changes (arrows)) of case #1. (C) Changes in hemoglobin (upper graph), creatinine, and erythropoietin (lower graph) concentrations in case #1. The upper panels show the serum ferritin level and the dosage of oral daprodustat administered in case #1.

Case #1 was a 72‐year‐old male who was referred to our department for the evaluation of anemia. He had past histories of hypertension and CKD and was treated with irbesartan and amlodipine for his hypertension which was under control. Complete blood count (CBC) revealed leukopenia (3.1 × 10^9^/L), anemia (hemoglobin, Hb, 110 g/L), and slight neutropenia (1.3 × 10^9^/L) with no circulating blasts. The bone marrow (BM) was normocellular with 1.6% myeloblasts. The BM smear presented with granulocytic dysplasia (decreased granules, nuclear hyposegmentation) (Figure 1A) and erythroid dysplasia (nuclear budding, karyorrhexis, multinuclearity, and megaloblastoid changes) (Figure 1B). Cytogenetic testing revealed a normal karyotype. Targeted deep sequencing (TDS) of BM sample using Illumina myeloid panel and institutional on‐demand panel which focus on 90 myeloid cancer‐related genes detected no pathogenic variants. Based on these results, he was diagnosed with MDS with multilineage dysplasia (MDS‐MLD) and was categorized as very low risk based on the revised international prognostic scoring system (IPSS‐R). Shortly after diagnosis, his anemia worsened to Hb 96 g/L. In parallel, he had renal insufficiency as evidenced by increased serum creatinine (1.41 mg/dL) with neither sign of bleeding nor decreased vitamin B12/folic acid levels. These results suggested that the worsening of anemia was largely caused by both MDS and renal anemia. Subsequently, oral administration of daprodustat 2 mg/day was started as a treatment for renal anemia (Figure 1C). Since the 6‐week administration of daprodustat 2 mg/day did not improve his anemia, we increased the dosage to 4 mg/day (Figure 1C). After dose escalation, his anemia significantly improved and he achieved hematological improvement‐erythroid (HI‐E) (Figure 1C) [11]. Concomitantly, his serum creatinine gradually increased whereas his serum EPO decreased (Figure 1C). Transformation into acute myeloid leukemia (AML) was not observed.

**FIGURE 2:**
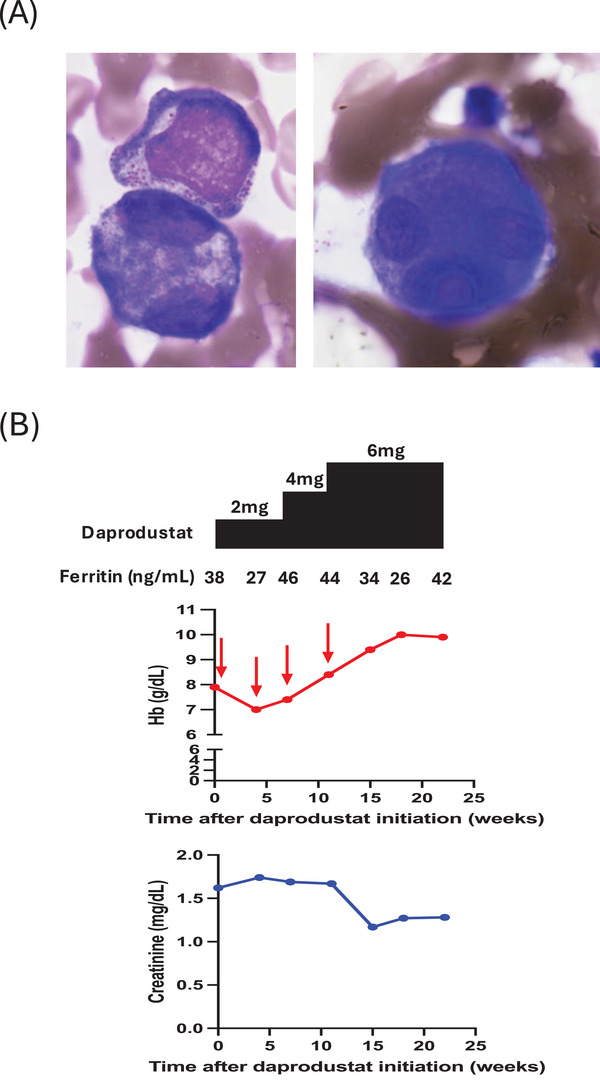
(A) Representative images of BM smears showing megakaryocytic dysplasia (left panel; micromegakaryocyte, right panel; multinucleation) of case #2. (B) Changes in hemoglobin (upper graph) and creatinine (lower graph) concentrations in case #2. Each red arrow depicts two units of red blood cell transfusion. The upper panels show the serum ferritin level and the dosage of oral daprodustat administered in case #2. EPO, erythropoietin; Hb, hemoglobin.

Case #2 was an 85‐year‐old female who was referred to our department for the evaluation of anemia. She had a past history of diabetes mellitus (DM) and was treated with glimepiride and linagliptin for her DM which was under control. CBC revealed leukopenia (2.6 × 10^9^/L), anemia (Hb 95 g/L), thrombocytopenia (95 × 10^9^/L), and slight neutropenia (1.3 × 10^9^/L) with no circulating blasts. The BM was normocellular with 1.4% myeloblasts. The BM smear presented with megakaryocytic dysplasia (micromegakaryocyte and multinucleation) (Figure 2A). Cytogenetic testing revealed a normal karyotype. TDS detected one variant in a gene encoding histone modifier KDM6A (*KDM6A* c.2194A > G, p.Arg732Gly), in which recurrent genetic mutations are reported in MDS [12]. Notably, *KDM6A* c.2194A > G was considered to be disease‐causing based on Mutation Taster [13]. Therefore, she was diagnosed with MDS with single lineage dysplasia (MDS‐SLD) and was categorized as low risk based on IPSS‐R. She was first treated with oral administration of CsA 150 mg/day and methenolone acetate (MA) 10 mg/day. However, she gradually developed CKD due to the side effects of CsA without improvement of her anemia, and therefore CsA and MA were discontinued. As her CKD persisted and anemia worsened to become a point of transfusion dependence (TD), she was started with oral administration of daprodustat 2 mg/day as a treatment for renal anemia (Figure 2B). Since the 7‐week administration of daprodustat 2 mg/day did not improve her anemia and TD, we increased the dosage to 4 mg/day (Figure 2B). 4‐week administration of daprodustat 4 mg/day was slightly effective for her anemia but still, the patient remained transfusion dependent (Figure 2B). After the dosage of daprodustat was increased to 6 mg/day, her anemia improved and she finally achieved transfusion independence (TI) and HI‐E (Figure 2B). Concomitantly, her serum creatinine gradually decreased (Figure 2B). Transformation into AML was not observed.

## 3 DISCUSSION

Here, we present two cases of low‐risk MDS complicated with CKD whose anemia responded to daprodustat treatment. In case #1, oral administration of daprodustat 4 mg/day significantly improved his anemia and the patient achieved HI‐E. Concomitantly, his serum creatinine gradually increased whereas his serum EPO decreased (Figure 1C). These data suggest that daprodustat‐induced anemia improvement was neither due to restoration of renal insufficiency nor serum EPO level. Rather, daprodustat may have restored anemia caused by MDS in this case. On the other hand, in case #2, the improvement of her anemia by daprodustat was accompanied by a gradual decrease in her serum creatinine (Figure 2B). Therefore, the daprodustat‐induced anemia improvement can be multifactorial in this case; restoration of renal anemia, MDS, or both.

Previous studies have shown potential efficacy of HIF‐PH inhibitors in MDS. Although the phase3 trial evaluating roxadustat for patients with transfusion‐dependent, lower‐risk MDS was terminated due to interim analysis outcomes not meeting statistical significance, a higher TI rate was achieved with roxadustat treatment compared with placebo, especially among MDS patients with a transfusion burden of two or more RBC units in 4 weeks [14]. Daprodustat, another HIF‐PH inhibitor, was reported to have a synergistic effect with dapagliflozin in cases with transfusion‐dependent MDS complicated with DM‐related CKD [15]. Together with the data shown in this report, these studies clearly indicate that HIF‐PH inhibitors can be a promising treatment option for MDS‐related anemia. Although these results are promising, whether daprodustat restored renal anemia or MDS‐related anemia could not be clearly determined. To this end, future clinical trials are required to explore if oral daprodustat alone is effective against MDS‐related anemia regardless of baseline renal function.

## AUTHOR CONTRIBUTIONS

Hiroyoshi Kunimoto and Hideaki Nakajima wrote the manuscript; Hiroyoshi Kunimoto developed figures; Hiroyoshi Kunimoto, Takayuki Sakuma, Takuma Ohashi, Mayoko Shirafuta, Hiroshi Teranaka, and Hideaki Nakajima carried out all the laboratory tests, were involved in patient management, and reviewed the manuscript.

## CONFLICT OF INTEREST STATEMENT

Hiroyoshi Kunimoto is a recipient of research funding from Daiichi‐Sankyo. Hideaki Nakajima is a recipient of research funding from Takeda, Astellas, Eisai, Pfizer, and Novartis.

## ETHICS STATEMENT

Approval was obtained from the Institutional Review Board at Yokohama City Hospital (approval #B240800062) and conducted in accordance with the Declaration of Helsinki protocol.

## PATIENT CONSENT STATEMENT

Patient consent was provided by the patient (both verbal and written).

## CLINICAL TRIAL REGISTRATION

The authors have confirmed clinical trial registration is not needed for this submission.

## Data Availability

The data that support the findings of this study are available from the corresponding author upon reasonable request.
